# Severe Pneumonia and Human Bocavirus in Adult

**DOI:** 10.3201/eid1210.060520

**Published:** 2006-10

**Authors:** Bernd Kupfer, Jörg Vehreschild, Oliver Cornely, Rolf Kaiser, Gerhard Plum, Sergei Viazov, Caspar Franzen, Ramona-Liza Tillmann, Arne Simon, Andreas Müller, Oliver Schildgen

**Affiliations:** * University of Bonn, Bonn, Germany;; † University of Cologne, Cologne, Germany;; ‡ Essen University Hospital, Essen, Germany;; § University of Regensburg, Regensburg, Germany

**Keywords:** bocavirus, respiratory, cancer, high-risk patients, letter

**To the Editor:** The newly identified human bocavirus (hBoV), a member of the *Parvovirus* family, is suspected to infect the cells of the respiratory tract and thus may be an etiologic agent of respiratory disease in humans (*1*). Although Koch postulates have not been fulfilled for HboV, it appears likely to cause a substantial number of respiratory tract infections, at least in children (*2**,**3*). We describe a case of severe atypical pneumonia associated with hBoV DNA in a bronchoalveolar lavage (BAL) sample from an adult.

The patient was a 28-year-old Caucasian woman with an angioimmunoblastic T–non-Hodgkin lymphoma (NHL) that changed into a highly malignant blastic B-cell lymphoma (T-cell–rich B-NHL state I with 70% CD20+ cells, initial stage IIIB). The patient was previously treated with vincristine and prednisone, followed by chemotherapy according to the R-CHOEP-14 protocol (3 cycles) (November 2003 through January 2004). From January through February 2004, chemotherapy was combined with antimicrobial drug therapy according to the R-DHAP protocol (which includes dexamethasone, the chemotherapy drugs cytarabine and cisplatin, and the monoclonal antibody drug rituximab) for persisting symptoms from the B-cell lymphoma. This regimen was followed by a therapy switch to alemtuzumab with ifosfamid, carboplatin, and etoposide (March 2004), which led to a therapy-induced leukopenia, thrombocytopenia, and high fever >40°C by the end of March and the beginning of April 2004. In May 2004, a second round of alemtuzumab with ifosfamid, carboplatin, and etoposide chemotherapy was initiated. In June 2004, a therapy-induced hemorrhagic cystitis occurred. During July 2004, the patient had ongoing high fever and aplasia of bone marrow with unclear etiology. On July 22, hospital treatment was initiated; it consisted of antimicrobial drug treatment with ceftriaxone (1,000 mg once daily) and gentamicin (320 mg once daily), and antimycotic therapy was started with caspofungin (50 mg once daily).

Since cytomegalovirus (CMV) infection was suspected, ganciclovir (250 mg twice daily) was administered IV for 2 weeks. Although the patient reported an ongoing cough and pneumonialike symptoms, a severe atypical pneumonia that was refractive to antibacterial and antimycotic treatment was diagnosed for the first time during this hospital treatment. Computed tomography scan showed bilateral atypical reticulonodular infiltrations predominant in the lower zones of the lungs (Figure).

**Figure:**
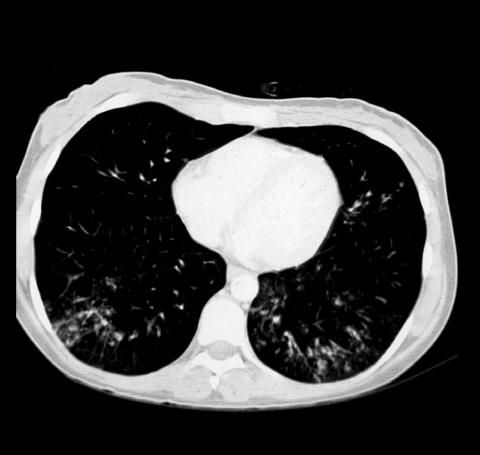
Computed tomography scan showing reticulonodular infiltrations of both lungs in the lower zones.

The BAL obtained during exacerbation of the pulmonary symptoms was tested for *Mycobacterium tuberculosis*, *Chlamydia pneumoniae*, *Pneumocystis jirovecii*, *Aspergillus* sp., *Candida* sp., *Cryptococcus neoformans*, CMV, Epstein-Barr virus, hepatitis B virus, hepatitis C virus, HIV, herpes simplex virus, and varicella-zoster virus by PCR and culture cultivation. Results were negative, except for a temporarily weak reactivity for *Aspergillus* antigen in serum and for CMV DNA in peripheral blood lymphocytes, which was positive before and became negative after ganciclovir therapy. An archived portion of the BAL was assayed retrospectively by PCR/reverse transcriptase–PCR for human bocavirus, respiratory syncytial virus, human coronaviruses including severe acute respiratory syndrome–associated coronavirus, influenza virus, and human metapneumovirus (hMPV). The only positive result was obtained for human bocavirus, which was confirmed by sequence analysis of the PCR product.

Within a few days, the patient's symptoms decreased, and she was discharged from hospital on day 41, despite ongoing bone marrow aplasia with antimicrobial and antimycotic prophylaxis, including trimethoprim/sulfamethoxazole (160 mg/800 mg once daily) and (voriconazole 200 mg twice daily). Clinical observations led to the primary assumption that the fever, cough, and pulmonary symptoms were likely caused by the postchemotherapeutic extended bone marrow aplasia and CMV infection accompanied by an unclear bacterial but fungus-typical infection. Retrospectively, however, human bocavirus DNA in the archived BAL strongly suggests that pulmonary symptoms were caused by this agent rather than by a yet unknown bacterial or fungal infection. Thus, in the clinical episode described here, the likely causative agent responsible for the severe pneumonia was the recently described bocavirus.

Respiratory viruses such as respiratory syncytial virus, hMPV, and hBoV seem to be the most prevalent etiologic agents of acute lower respiratory tract infection in children. Recently, evidence of human bocavirus infection was reported for 3.1% to 5.7% of children <3 years of age (*1**–**3*). Previously, only limited data on adults, including immunocompromised patients, were available, but the case we describe supports the hypothesis proposed for other newly identified respiratory viruses, namely, that these pathogens also contribute to severe infections in adult patients at high risk. For example, hMPV was found in 3% of stem-cell transplant recipients who underwent BAL because of lower respiratory tract infection (*4*). In those high-risk patients, hMPV also induced fatal infections (*4*). This finding led to the conclusion that a "new" virus that induces the identical clinical symptoms, like the human bocavirus, may also contribute to severe respiratory infections. In summary, this first report of a respiratory tract infection with hBoV in an adult immunocompromised patient strongly supports the assumption that hBoV is an emerging pathogen that requires our attention, even for adult patients (*1**–**3*).
